# Selected Amino Acids Promote Mouse Pre-implantation Embryo Development in a Growth Factor-Like Manner

**DOI:** 10.3389/fphys.2020.00140

**Published:** 2020-03-10

**Authors:** Michael B. Morris, Sukran Ozsoy, Matthew Zada, Mark Zada, Radu C. Zamfirescu, Mariana G. Todorova, Margot L. Day

**Affiliations:** ^1^Discipline of Physiology, School of Medical Sciences, The University of Sydney, Sydney, NSW, Australia; ^2^Bosch Institute, The University of Sydney, Sydney, NSW, Australia

**Keywords:** amino acids, L-Proline, L-Glutamine, pre-implantation embryo, blastocyst development, hatching, low-density culture

## Abstract

Groups of amino acids, and some selected amino acids, added to media used for culture of pre-implantation embryos have previously been shown to improve development in various ways including survival to the blastocyst stage, increased blastocyst cell number and improved hatching. In this study, we cultured 1-cell mouse embryos for 5 days to the hatching blastocyst stage in isosmotic medium (270 mOsm/kg) at high density (10 embryos/10 μL), where autocrine/paracrine support of development occurs, and low density (1 embryo/100 μL), where autocrine/paracrine support is minimized and development is compromised. When 400 μM L-Pro or 1 mM L-Gln was added to embryos at low density, the percentage of embryos reaching the blastocyst stage and the percentage hatching increased compared to low-density culture without these amino acids, and were now similar to those for embryos cultured at high density without amino acids. When L-Pro or L-Gln was added to embryos at high density, the percentage of embryos reaching the blastocyst stage didn’t change but hatching improved. Neither embryo culture density nor the presence of these amino acids had any effect on blastocyst cell number. D-Pro and the osmolytes Gly and Betaine did not improve embryo development in low- or high-density culture indicating the mechanism was stereospecific and not osmotic, respectively. L-Pro- and L-Gln-mediated improvement in development is observed from the 5-cell stage and persists to the blastocyst stage. Molar excess of Gly, Betaine or L-Leu over L-Pro eliminated improvement in development and hatching consistent with them acting as competitive inhibitors of transporter-mediated uptake across the plasma membrane. The L-Pro effect is dependent on mTORC1 signaling (rapamycin sensitive) while that for L-Gln is not. The addition of L-Pro leads to significant nuclear translocation of p-Akt^S473^ at the 2- and 4-cell stages and of p-ERK1/2^T202/Y204^ nuclear translocation at the 2-, 4-, and 8-cell stages. L-Pro improvement in embryo development involves mechanisms analogous to those seen with Pro-mediated differentiation of mouse ES cells, which is also stereoselective, dependent on transporter uptake, and activates Akt, ERK, and mTORC1 signaling pathways.

## Introduction

Groups of amino acids and some selected amino acids can improve *in vitro* development of cultured pre-implantation mammalian embryos from a variety of species (Ho et al., 1995). Non-essential amino acids are thought to promote development up to the ∼8–16 cell stage. From then on, essential amino acids stimulate the development of the inner cell mass, while non-essential amino acids stimulate blastocyst expansion and hatching (Lane et al., 2001). For mammalian embryos, the two-stage or sequential media (e.g., G1/G2 media for human embryos) can be used to take advantage of this to promote the viability and health of cultured embryos and improve the prospects of live births following implantation (Steeves and Gardner, 1999; Gardner and Lane, 2003; Summers and Biggers, 2003).

L-Gln is the most studied of the individual amino acids. Its presence can be toxic to embryos due to the spontaneous and metabolic generation of ammonium. However, if ammonium is removed from the medium, either by replacing the medium or by substituting L-Gln with a dipeptide form [either Alanyl-L-Gln (Lane et al., 2001) or Glycyl-L-Gln (Biggers et al., 2004)] then L-Gln promotes development over the 2- to 8-cell stages in a number of species (Chatot et al., 1989; Erbach et al., 1994; Lane and Gardner, 1997).

More recently, selected amino acids including L-Pro, Betaine, and Gly have been shown to act as osmolytes for mouse embryos, which protect against the detrimental effects of culture in hyperosmotic conditions (>300 mOsm/kg) (Baltz and Tartia, 2010). In particular, the SIT1 transporter (Slc6a20; SIT1 or IMINO), which is expressed at the 1- to 2-cell stage, has been implicated in the uptake of these osmolytes (Anas et al., 2008). This Na^+^-amino acid co-transporter allows for millimolar accumulation of Betaine and L-Pro in the embryo over and above the extracellular concentration. Similarly, the GLYT1 transporter, which is expressed until compaction (Van Winkle et al., 1988), allows uptake of Gly sufficient to curb the inhibitory effects of hyperosmolarity on mouse embryo development (Steeves et al., 2003).

Aside from this straightforward osmotic effect under hyperosmotic conditions, the mechanisms relating to improved development of embryos in the presence of amino acids under isosmotic conditions remain obscure. In particular, little if any consideration has been given to the following: Which amino acids are bioactive and improve development? Which antagonize the effects of those that are bioactive? Is bioactivity dependent on the culture density of embryos? With respect to the last question, mouse embryos provide autocrine/paracrine support for development when cultured at high density (HD) (e.g., 10 embryos/10 μL) and this effect is mitigated in low-density (LD) culture (e.g., 1 embryo/100 μL) (Lane and Gardner, 1992; O’Neill, 1997; Green and Day, 2013). A number of autocrine/paracrine embryotrophic factors have been identified, including PAF and IGF1. PAF stimulates intracellular Ca^2+^ transients (Li et al., 2007), which activate Ca^2+^-sensitive Cl^–^ channels (Li et al., 2007, 2009). Both PAF and IGF1 decrease apoptosis by activating the PI3K pathway (Jin et al., 2009; Green and Day, 2013) thereby improving embryo development.

Here we show that when selected amino acids, L-Pro or L-Gln, are added to isosmotic medium, development to the blastocyst stage is improved when embryos are cultured at LD but not at HD, suggesting they act as effective replacements of autocrine/paracrine support. Neither L-Pro or L-Gln increase cell number in blastocysts, consistent with the effect not being a simple nutritional one. Selected amino acids added in molar excess inhibit the effect of L-Pro and in some cases reduce development on their own, indicating that the indiscriminate addition of groups of amino acids to the culture medium may be counterproductive. L-Pro stimulates nuclear translocation of p-Akt^S473^ and p-ERK1/2^T202/Y204^ and the improvement in development is dependent on mTORC1 signaling. These results are analogous to L-Pro’s growth-factor-like stimulation of differentiation of mouse ES cells, which is also dependent on transporter-mediated uptake and involves activation of the Akt, ERK and mTORC1 signaling pathways (Lonic, 2007; Washington et al., 2010; Tan et al., 2011).

## Materials and Methods

### Mice, Zygote Isolation and Culture

Outbred Quackenbush Swiss (QS) mice (Animal Research Centre, Perth, Australia and Laboratory Animal Services, The University of Sydney) were used in accordance with the Australian Code of Practice for Use of Animals in Research and the study was approved by the University of Sydney Animal Care and Ethics Committee (approval numbers 4838, 5583, and 824). Mice were housed under a 12-h light:12-h dark cycle. Female mice (4-10 weeks old) were induced to ovulate by intraperitoneal injection of 10 IU pregnant mare serum gonadotrophin (PMSG; Intervet) followed 48 h later by 10 IU human chorionic gonadotrophin (hCG) and paired with males overnight. Female mice were euthanized by cervical dislocation 20-22 h after hCG injection. Zygotes were isolated as described previously (Green and Day, 2013). The medium used for isolation of zygotes was Hepes-buffered modified synthetic human tubal fluid (Hepes-mHTF (Quinn, 1995)), in which the concentration of NaCl was adjusted to 112 mM, 102 mM or 85 mM in order to achieve a final osmolality of 330, 300 or 270 mOsm/kg, respectively. Medium with an osmolality of 270 mOsm/kg was considered isosmotic with the intracellular fluid (Baltz, 2001). A reduced amount of bovine serum albumin (BSA; 0.3 mg/mL) was added to all media to minimize the possibility that break down of BSA into free amino acids could contribute to the final amino acid concentration in the medium.

Embryo culture was performed in mHTF containing 0.3 mg/mL BSA and with the different NaCl concentrations and osmolalities described above. L-Gln was omitted from all media unless stated. Embryos were cultured from the zygote stage (day 1 of development) for 120 h (humidified incubator at 37°C and 5% CO_2_) to day 6 of development, either in dishes (for HD culture) or round bottom, 96-well plates (for HD and LD culture) (Corning, NY, United States). HD culture was performed by placing 10 embryos into a 10 μL drop of medium under mineral oil (Sigma). LD culture was performed by placing individual embryos into 100 μL medium in each well of a 96-well plate. Medium was pre-equilibrated at 37°C with 5% CO_2_ for a minimum of 2 h before adding the embryos. Embryos were scored for developmental stage every 24 h. Hatching from the *zona pellucida* was scored on day 6 of development (144 h post-hCG). The medium was not changed over the culture period unless indicated.

### Culture of Embryos in Amino Acids

Amino acids were prepared as either 1 M, 250 or 100 mM stock solutions in water, sterilized by filtration and stored at −20°C. Embryo culture was performed in final concentrations of amino acids of either 0.4 mM or 1 mM, as indicated. When amino acids were used as competitive inhibitors of L-Pro uptake they were used at a final concentration of 5 mM.

### Cell Number Determination

Total cell numbers in blastocysts were analyzed by staining with DAPI. Blastocysts were fixed on day 6 of development by incubation in 4% paraformaldehyde (PFA) in PBS/polyvinyl alcohol (1 mg/mL PVA, #H-1200) for 30 min, followed by permeabilization in PBS/PVA + 0.3% Triton X-100 for 30 min at RT. Blastocysts were then mounted on coverslips in Vectashield + DAPI (Vector Labs) and imaged by confocal microscopy (Zeiss LSM510 Meta). The number of nuclei stained with DAPI was counted to give the total cell number.

### Immunofluorescent Staining of p-Akt^S473^ and p-ERK1/2^T202/Y204^

After being cultured in various treatments to the 2-, 4- or 8-cell stages, embryos were fixed in 4% PFA in PBS/PVA for 30 min at room temperature and then washed twice in PBS/PVA.

In order to eliminate variability between samples during phosphoprotein staining, embryos in different treatment groups underwent the staining process together. To achieve this, the membranes of embryos cultured in control medium were first labeled with concanavalin A Alexa Fluor 647 (ConA; Invitrogen Molecular Probes, #C21421) (1:50 dilution in PBS/PVA) for 30 min in the dark at room temperature. Then, embryos in control and treatment groups underwent the rest of the staining process together in the same aliquot of phospho-antibody and were identified later by the presence or absence of ConA staining.

Embryos were permeabilized in PBS/PVA + 0.3% Triton X-100 for 30 min at room temperature, then washed in PBS/PVA before being blocked in PBS/PVA + 0.7% BSA + 0.1% Tween-20 for 30 min at room temperature. Primary and secondary antibodies were diluted in PBS + PVA + 0.7% BSA + 0.1% Tween-20, as follows: 1:200 for p-ERK^T202/Y204^ (Cell Signaling, #9101), 1:50 for p-Akt^S473^ (Cell Signaling, #4058), 1:200 for goat anti-rabbit IgG Alexa Fluor 488 (Molecular Probes, #A-11008). For primary antibodies, embryos were incubated overnight at 4°C and then washed three times in PBS/PVA + 0.7% BSA + 0.1% Tween-20. Embryos were then incubated in secondary antibody in the dark at room temperature for 2 h and again washed three times in PBS + PVA + 0.7% BSA + 0.1% Tween-20. Embryos were transferred to a drop of Vectashield + DAPI and DAPI-stained nuclei and secondary antibody fluorescence were visualized on a confocal microscope with a 40× objective using 405 and 488 nm Argon lasers, respectively. ConA fluorescence was visualized using a helium-neon laser at 633 nm. Embryos were scanned through the plane of the nuclei for the purpose of measuring the relative fluorescence intensity in the nuclei compared to the cytoplasm using ImageJ software.

For each blastomere, average nuclear fluorescence intensity was determined from independent measurements in three square areas of the same size excluding the nucleoli. The same measurements were performed in the cytoplasm. The ratio of the average nuclear intensity to the average cytoplasm intensity was calculated to indicate the extent of nuclear phosphoprotein localization. Mean intensity measurements were performed blind by randomization of files.

### Statistical Analysis

Culture experiments were performed at least three times, with at least 15 embryos in each treatment group, and the data pooled. Proportions were compared using Fisher’s exact test or Chi-squared analysis, with Bonferonni corrected significance levels for multiple comparisons. Cell counts from at least three independent experiments were pooled and expressed as mean ± SEM and data were compared using ANOVA, as described in the figure legends, using Graphpad Prism v7. Fluorescent nuclear/cytoplasmic ratios were pooled from at least three independent experiments and data were compared using an unpaired *t*-test.

## Results

### LD Culture Compromises Pre-Implantation Development

**FIGURE 1:**
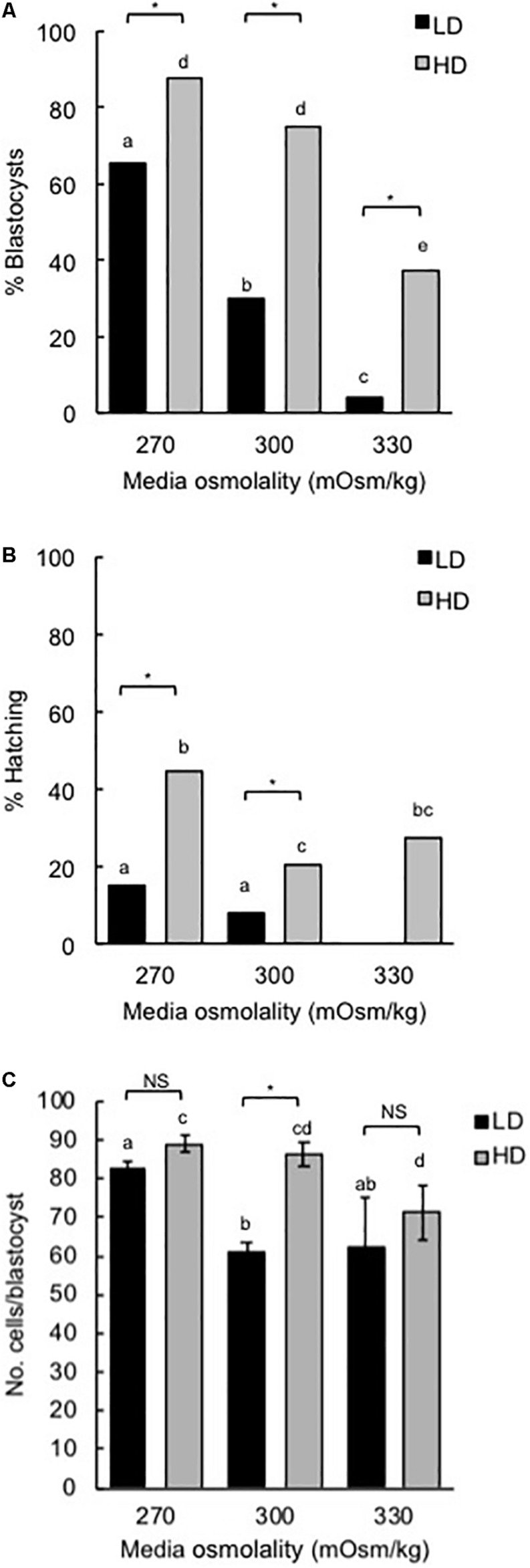
Effect of culture density and media osmolality on the development of mouse zygotes to the blastocyst stage. Zygotes (20–22 h post-hCG) were cultured in media of different osmolality for 5 days at LD (1 embryo/100 μL) or HD (10 embryo/10 μL). **(A)** Percentage of zygotes that developed to the blastocyst stage. **(B)** Percentage of hatching blastocysts. **(C)** Cell numbers in blastocysts (mean ± SEM). Embryo development was analyzed from 9 independent experiments with 15 embryos per treatment in each experiment. *P < 0.05; NS, not significant. Bars with different letters within the same culture density are significantly different. Proportions in panels **(A,B)** were compared using Fisher’s exact test, with Bonferroni correction for multiple comparisons, and means in panel **(C)** compared using two-way ANOVA with Sidak’s multiple comparison test.

Media with an osmolality of 270 mOsm/kg was used in this study as it resulted in optimal blastocyst development and blastocyst hatching in both HD (10 embryos/10 μL) and LD (1 embryo/100 μL) culture conditions (Figure 1). Mouse zygotes cultured at LD without amino acids for 5 days in 270 mOsm/kg mHTF medium had reduced development (% blastocysts formed) and hatching compared to embryos cultured at HD (Figures 1A,B). This difference is believed to occur because the development of embryos cultured at HD is supported by autocrine/paracrine factors (Lane and Gardner, 1992; O’Neill, 1997; Green and Day, 2013). On the other hand, there was no difference in the number of cells in blastocysts developed in 270 mOsm/kg medium at LD or HD (Figure 1C).

For LD at higher osmolality (300 and 330 mOsm/kg), development to the blastocyst stage and hatching were reduced. HD culture was able to partially overcome these effects (Figures 1A,B). Similarly, cell numbers in those blastocysts that did develop in 300 mOsm/kg medium at LD were reduced compared to embryos developed in 270 mOsm/kg and this effect was negated by HD culture (Figure 1C).

### L-Pro and L-Gln Improve Blastocyst Development and Hatching in LD Culture

**FIGURE 2:**
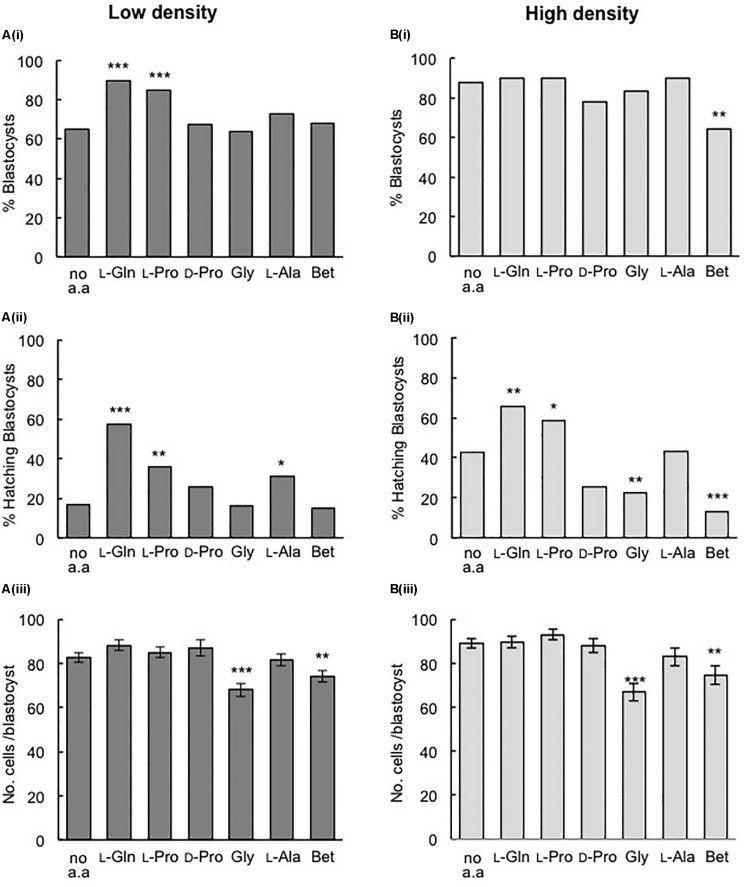
Effect of individual amino acids on development of mouse embryos cultured in isosmotic (270 mOsm/kg) medium at LD **(Ai–iii)** and HD **(Bi–iii).** Zygotes (20–22 h post-hCG) were cultured for 5 days in the absence of amino acids (no a.a.) or in the presence of 1 mM L-Gln, 400 μM L-Pro, 400 μM D-Pro, 400 μM Gly, 400 μM L-Ala or 1 mM Betaine (Bet). **(i)** Percentage of zygotes that developed to the blastocyst stage. **(ii)** Percentage of hatching blastocysts. **(iii)** Cell numbers in blastocysts (mean ± SEM, *n* = 25–94). Embryos were analyzed from at least three independent biological replicates, each with at least 15 embryos per treatment. *P < 0.05; **P < 0.01; ***P < 0.001 indicates significant differences from the no amino acid control, analyzed by either Chi-squared test **(Ai,ii, Bi,ii)** or one-way ANOVA with Dunnett’s *post hoc* test **(Aiii, Biii)**.

For embryos cultured at LD in 270 mOsm/kg medium, the addition of either 400 μM L-Pro or 1 mM L-Gln increased the percentage of blastocysts formed and the percentage of blastocysts that hatched compared to embryos grown in the absence of amino acids (Figures 2Ai,Aii). In fact, the values increased to those similar to that seen in HD culture in the absence of amino acids (Figures 2Bi,Bii). However, neither L-Pro nor L-Gln increased the average number of cells in blastocysts compared to embryos cultured without amino acids (Figures 2Aiii,Biii).

A range of other amino acids – the D-isomer of Proline (D-Pro), L-Ala, and the osmolytes Gly and Betaine – failed to increase blastocyst development in LD culture at 270 mOsm/kg (Figure 2Ai) and all except L-Ala failed to improve hatching (Figure 2Aii). As with L-Pro and L-Gln, none of the other amino acids increased blastocyst cell number (Figure 2Aiii) in LD culture, while Gly and Betaine actually decreased blastocyst cell number (Figure 2Aiii).

### L-Pro and L-Gln Improve Hatching but Not Blastocyst Development in HD Culture

In HD culture at 270 mOsm/kg, none of the amino acids improved blastocyst development or increased cell numbers compared to embryos cultured without amino acids (Figures 2Bi,Biii). However, L-Pro and L-Gln improved hatching at HD (Figure 2Bii), as they did in LD culture. Betaine decreased blastocyst development and both Gly and Betaine also decreased blastocyst hatching and blastocyst cell numbers.

### All Added Amino Acids Improve Blastocyst Development Under Hyperosmotic Conditions

**FIGURE 3:**
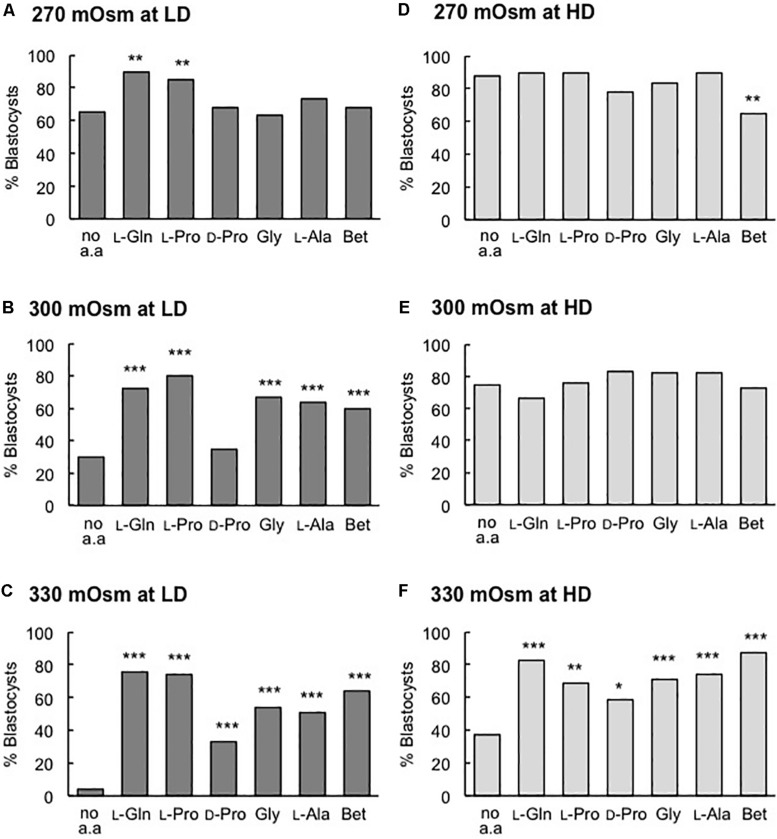
Effect of individual amino acids on blastocyst development in media of different osmolality at LD **(A–C)** and HD **(D–F)**. Zygotes (20–22 h post-hCG) were cultured for 5 days in the absence of amino acids (no a.a.) or in the presence of 1 mM L-Gln, 400 μM L-Pro, 400 μM D-Pro, 400 μM Gly, 400 μM L-Ala or 1 mM Betaine (Bet) in medium with an osmolality of **(A,D)** 270 mOsm/kg, **(B,E)** 300 mOsm/kg and **(C,F)** 330 mOsm/kg. Embryos were analyzed from at least three independent biological replicates, each with at least 15 embryos per treatment. *P < 0.05; **P < 0.01; ***P < 0.001 indicates significant differences from the no amino acid control, analyzed by Chi-squared test.

As noted previously, in LD culture under isosmotic conditions (270 mOsm/kg), only added L-Pro or L-Gln increased the percentage of blastocysts that formed (Figures 2Ai,3A), and no improvement with these amino acids was observed at HD (Figures 2Bi, 3D). Under increasingly hyperosmotic conditions (300, then 330 mOsm/kg), three changes occurred compared to results obtained under isosmotic conditions: (i) The percentage of embryos that developed to the blastocyst stage progressively decreased in both LD and HD culture in the no amino acid controls (Figure 1, and compare Figures 3B,C with Figure 3A, and Figures 3E,F with Figure 3D). (ii) This loss of viability at LD was partially or completely reversed by any of the added amino acids (Figures 3B,C). (iii) HD culture was able to overcome the loss of viability seen with LD culture at 300 mOsm/kg (Figure 3E) but addition of amino acids was also required to overcome the loss at 330 mOsm/kg medium (Figure 3F). These results are consistent with data showing that a large range of amino acids can act as osmolytes to offset the damaging effects of hyperosmolality (Steeves et al., 2003; Anas et al., 2008; Baltz and Tartia, 2010).

### L-Pro and L-Gln Improve Blastocyst Development in LD Culture After the 2-Cell Stage

**FIGURE 4:**
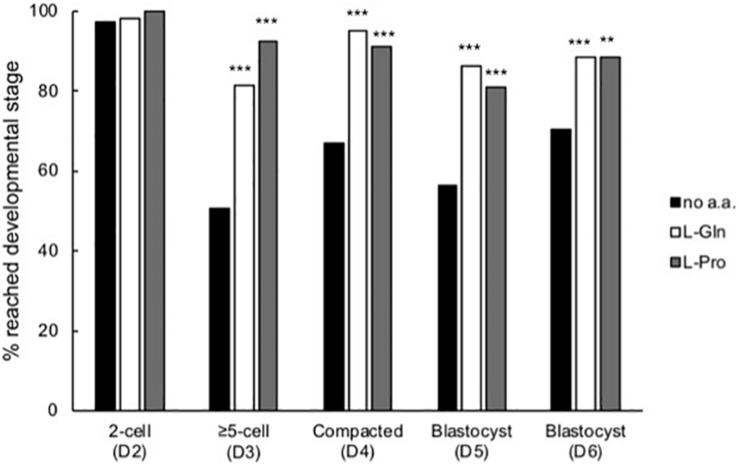
Time-dependent effect of L-Pro and L-Gln on development of embryos cultured in isosmotic (270 mOsm/kg) medium at LD. Zygotes (20–22 post-hCG, D1 of development) were cultured for up to 5 days either in the absence of amino acids (no a.a.) or in the presence of 1 mM L-Gln or 400 μM L-Pro and scored for development to the appropriate stage over the 5 days of culture. D indicates the day of development. A total of 79–158 individual embryos were analyzed from at least four independent biological replicates. **P < 0.01; ***P < 0.001 indicates significant differences from the no amino acid control at each time point, analyzed by Chi-squared test.

Zygotes cultured at LD in isosmotic medium were scored for stages of development each day over the 5-day culture period (Figure 4). Almost all zygotes progressed to the 2-cell stage both in the absence and presence of amino acids. By the third day (D3), embryos cultured in the presence of L-Pro or L-Gln showed improved development compared to embryos cultured without amino acids and this improvement was maintained for the remainder of the culture period (Figure 4). None of the other amino acids tested showed improved development over the no amino acid control at any stage (data not shown).

**FIGURE 5:**
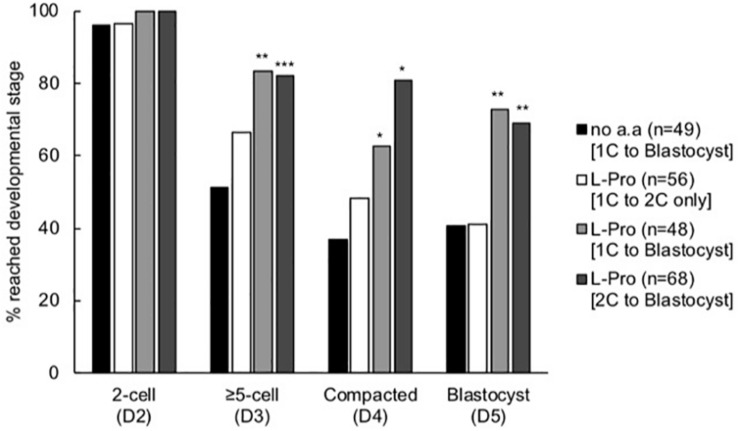
Effect of timing of addition of L-Pro on development of mouse embryos cultured in isosmotic (270 mOsm/kg) medium at LD. Zygotes (20–22 h post-hCG) were cultured for 5 days in the absence of amino acids (no a.a. [1C to blastocyst]) or in the presence of 400 μM L-Pro (i) for the first 24 h of culture (i.e., from the 1-cell (1C) to 2-cell (2C) stages) and then transferred to no a.a. medium for the remainder of the culture period (L-Pro [1C to 2C only]); (ii) for the full 5 days (L-Pro [1C to blastocyst]); or (iii) from the 2-cell stage onward following 24 h culture in no a.a. (L-Pro [2C to blastocyst]). The percentage of zygotes that developed to the designated stages over the 5 days was recorded. D indicates the day of development; *n* = number of individual embryos that were analyzed from at least four independent biological replicates. *P < 0.05; **P < 0.01; ***P < 0.001 indicates significant differences from the no amino acid control at each time point, analyzed by Chi-squared test.

The timing of the beneficial effect of L-Pro on development was investigated by culturing embryos in the presence of L-Pro over restricted time windows. Embryos cultured in the presence of L-Pro only for the first 24 h (up until the late 2-cell stage) developed no better than embryos cultured in the absence of amino acids (Figure 5). In contrast, embryos cultured in the presence of L-Pro only from the late 2-cell stage onward had improved development similar to that which occurred when L-Pro was present throughout the culture period, from the 1-cell stage to the blastocyst (Figure 5).

### Molar Excess of L-Leu, Gly or Betaine Inhibits L-Pro-Mediated Development in LD Culture

**FIGURE 6:**
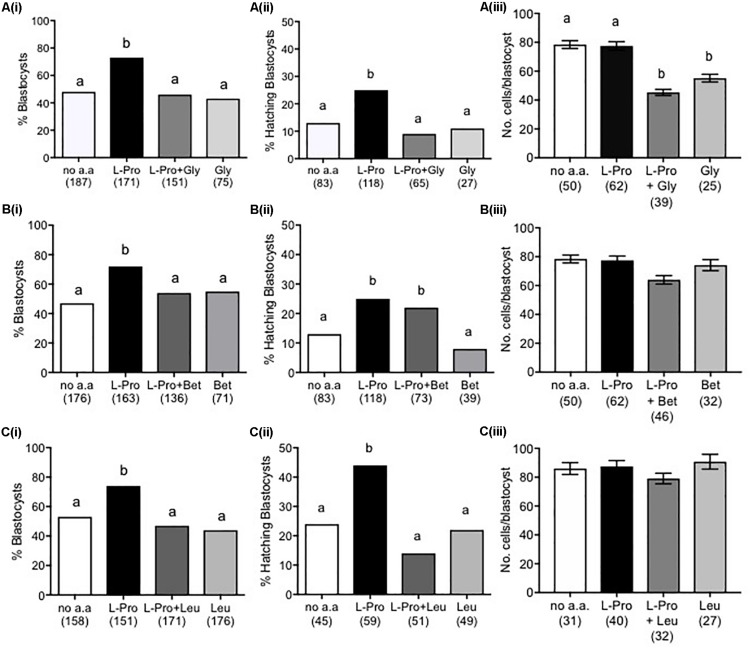
Effect of molar excess of Gly, Betaine and L-Leu on the L-Pro-mediated improvement in blastocyst development of mouse embryos cultured in isosmotic (270 mOsm/kg) medium at LD. Zygotes (20–22 h post-hCG) were cultured for 5 days in no amino acids (no a.a.), 400 μM L-Pro alone or 400 μM L-Pro in the presence of **(A)** 5 mM Gly, **(B)** 5 mM Betaine or **(C)** 5 mM L-Leu. **(i)** Percentage of blastocyst development. **(ii)** Percentage of blastocysts hatching. **(iii)** Number of cells in blastocysts. Values in **(iii)** are mean ± SEM. The number of embryos analyzed from at least three separate experiments is given in parentheses. Bars with different letters are significantly different (*P* < 0.05) using Fisher’s exact test, with Bonferroni correction for multiple comparisons, and one-way ANOVA with Tukey’s *post hoc* test for mean values.

We have previously shown that L-Pro induces the differentiation of mouse embryonic stem cells (ESCs) to primitive ectoderm and that this differentiation can be inhibited by the addition of molar excess of selected amino acids, which competitively inhibit plasma-membrane transport via the amino-acid transporter SNAT2 (Washington et al., 2010; Tan et al., 2011). Similarly, L-Pro may mediate improved embryo development via amino-acid transporter(s). Consistent with this, the addition of 5 mM L-Leu, Gly or Betaine prevented L-Pro-mediated improvement in embryo development to the blastocyst stage, while none of L-Leu, Gly or Betaine added on its own had any effect (Figures 6Ai–Ci). In terms of hatching, both Gly and L-Leu inhibited L-Pro-mediated improvement but, unlike development to the blastocyst stage, Betaine did not (Figures 6Aii–Cii). The presence of 5 mM Gly decreased cell numbers in blastocysts (Figure 6Aiii), while 5 mM Betaine or L-Leu had no effect on cell numbers (Figures 6Biii,Ciii).

### Inhibition of the mTORC1 Pathway Prevents L-Pro-Mediated Development in LD Culture

**FIGURE 7:**
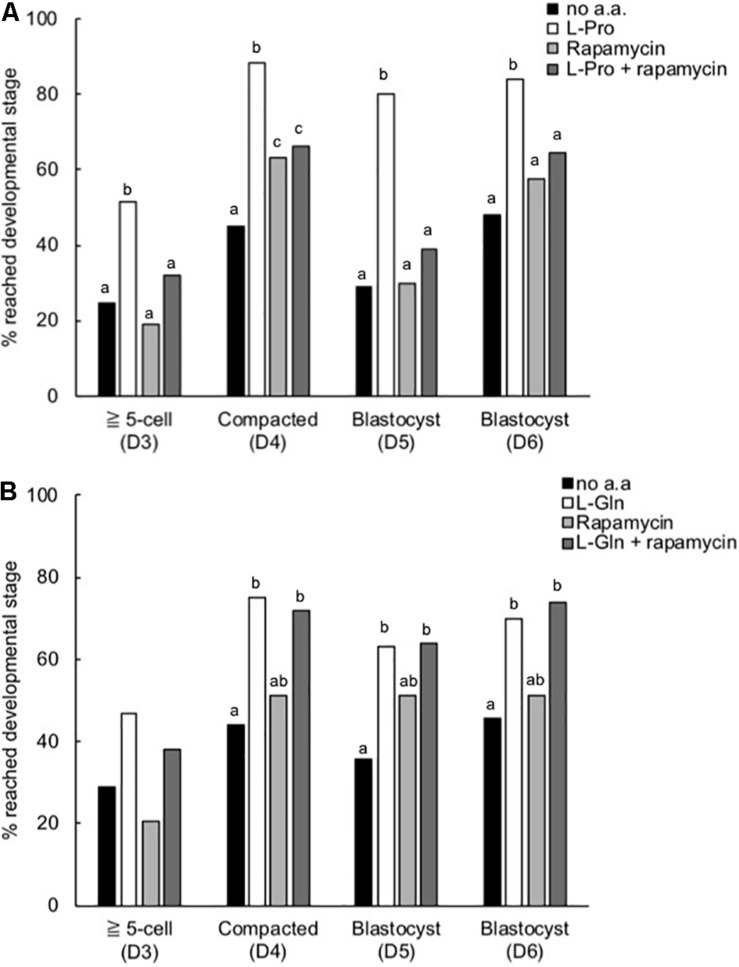
Effect of rapamycin on the L-Pro- and L-Gln-mediated improvement in development of mouse embryos cultured in isosmotic (270 mOsm/kg) medium at LD. Zygotes (20–22 h post-hCG) were cultured for 5 days in no amino acids (no a.a.), 400 μM L-Pro alone **(A)**, 1 mM L-Gln alone **(B)**, 10 nM rapamycin alone or 10 nM rapamycin in the presence of **(A)** 400 μM L-Pro or **(B)** 1 mM L-Gln. The percentage of zygotes that developed to the designated stages over the 5 days was recorded. D indicates the day of development; embryos were analyzed from at least three independent biological replicates. Bars with different letters are significantly different within each day of development, determined using Fisher’s exact test, with Bonferroni correction for multiple comparisons (*P* < 0.0125).

When the highly selective mTORC1 inhibitor rapamycin (10 nM) was added to embryos in culture from the 2-cell stage, the L-Pro-mediated improvement in development to the blastocyst stage was prevented (Figure 7A). At this concentration, rapamycin itself has no effect on development. In contrast, rapamycin did not inhibit the L-Gln-mediated improvement in development (Figure 7B).

### L-Pro Stimulates Nuclear Translocation of p-Akt^S473^ and p-ERK^T202/Y204^

**FIGURE 8:**
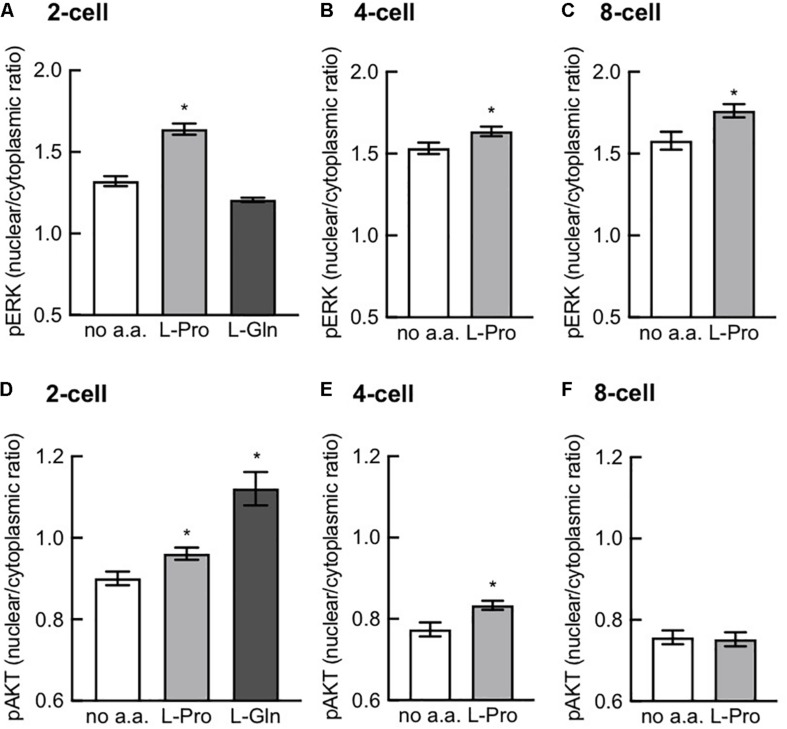
Effect of L-Pro and L-Gln on nuclear translocation of p-ERK^T202/Y204^ and p-Akt^S473^. Zygotes (20–22 h post-hCG) were cultured for 2–3 days in no amino acids (no a.a.), 400 μM L-Pro or 1 mM L-Gln and then fixed and immunostained for p-ERK^T202/Y204^ **(A–C)** and p-Akt^S473^ **(D–F)**. Intensity of fluorescence in the nucleus and cytoplasm was measured and the nuclear-to-cytoplasmic ratio was calculated in 2-, 4-, and 8-cell embryos for L-Pro and in 2-cell embryos for L-Gln. Embryos were analyzed from at least three independent biological replicates with 5–10 embryos per experiment. Values are mean ± SEM. *P < 0.05 using an unpaired *t*-test **(B,C,E,F)** or one-way ANOVA with Dunnett’s *post hoc* test **(A,D)**.

Figure 8 shows that L-Pro added to zygotes stimulated nuclear translocation of p-Akt^S473^ at the 2- and 4-cell stages but not at the 8-cell stage, while nuclear translocation of p-ERK^T202/Y204^ was increased at all 3 of these stages of development. L-Gln added to zygotes also stimulated nuclear translocation of p-Akt^S473^ at the 2-cell stage but had no effect on nuclear translocation of p-ERK^T202/Y204^. Further investigation is needed to confirm whether L-Gln can act in a growth factor like manner in pre-implantation embryos.

## Discussion

Our results show that L-Pro and L-Gln each improves the development of embryos to the blastocyst stage when embryos are cultured at LD (Figure 2A). The effect is stereoselective (D-Pro has no effect) and is not a simple osmotic one since (i) it occurs in isosmotic medium, (ii) does not occur when embryos are cultured at HD (Figure 2B) and (iii) known osmolytes such as Betaine and Gly (Baltz and Tartia, 2010) failed to improve development in this isosmotic medium either in LD or HD culture (Figures 2Ai,Bi). Osmolytes do, however, as expected (Baltz and Tartia, 2010), improve development to the blastocyst stage at 330 mOsm/kg in HD culture (Figure 3F) and at ≥300 mOsm/kg at LD (Figures 3B,C). Nor does the improvement in blastocyst development with L-Pro or L-Gln under isosmotic conditions at LD appear to be a simple nutritional effect: Neither L-Pro nor L-Gln increased blastocyst cell number in LD (or HD) culture (Figures 2Aiii, 2Biii). Increasing culture time from 5 to 6 days did not increase the number of blastocysts in LD culture suggesting this is not a kinetic effect resulting from delayed development in the absence of these amino acids or more rapid development in their presence (data not shown).

Instead, our data suggest that L-Pro or L-Gln added to embryos cultured at LD under isosmotic conditions act in a growth factor- or autocrine-like manner to improve development. Consistent with this, the improvement in the development of embryos in LD culture in the presence of either of these specific amino acids was similar to that seen at HD in the absence of amino acids, where autocrine/paracrine support is available (Figure 2). A number of factors are known to provide autocrine/paracrine support for developing embryos including PAF and growth factors such as IGF1 (Li et al., 2007; Jin et al., 2009; Green and Day, 2013). Their collective presence and concentrations in HD culture may obviate the need for the exogenous addition of specific amino acids. However, secretion of non-essential amino acids such as L-Pro and L-Gln in pre-implantation embryos does occur (Van Winkle and Dickinson, 1995; Li et al., 2006) and we speculate that their endogenous production and release in HD culture contributes to the pool of autocrine/paracrine support along with previously identified factors such as PAF and IGF1.

*In vivo*, the developing embryo, as well as secreting its own amino acids, is bathed first in tubal and then uterine fluid. The concentrations of amino acids in these fluids fall in the low millimolar to submillimolar range; i.e., similar to the concentrations of L-Pro and L-Gln used here (0.4 and 1 mM, respectively) (Miller and Schultz, 1987; Aguilar and Reyley, 2005; Li et al., 2006; Kermack et al., 2015). Thus, amino-acid-mediated development of the pre-implantation embryo *in vivo* may result from amino acids derived from both autocrine/paracrine and maternal sources. L-Pro and L-Gln are conditionally essential amino acids; i.e., they can be produced endogenously but not necessarily at concentrations required to meet specific developmental milestones (Wu, 2013; Wu et al., 2013). Sufficient concentrations of L-Gln and/or L-Pro in the maternal environment (e.g., in the tubal fluid) may be indicators of normal nutritional balance and thereby permit pregnancy/embryo development to continue.

Analogous to this, ESCs require medium supplementation with L-Gln to permit proliferation (Carey et al., 2015) and L-Pro to permit differentiation (Washington et al., 2010; Casalino et al., 2011; Shparberg et al., 2019a), even though both are produced endogenously. In the case of L-Pro, ESCs self-limit production by an autoregulatory loop mediated by free prolyl-tRNA (i.e., tRNA not loaded with proline) coupled to the amino-acid starvation response (AAR) pathway (D’Aniello et al., 2015). Exogenous addition of L-Pro overwhelms this autoregulation, thereby permitting directed lineage progression. It’s not known if this autoregulatory loop controls pre-implantation embryo development but other L-Pro-mediated signaling mechanisms we have observed here for embryos are analogous to those seen in L-Pro-mediated differentiation of ESCs, including stereoselectivity (Figure 2), activation of Akt and ERK signaling (Figure 8) and dependence on mTORC1 signaling (Figure 7; Lonic, 2007; Washington et al., 2010; Tan et al., 2011). We note that *mTOR*^–/–^ mouse embryos exhibit reduced proliferation and differentiation of the inner cell mass and trophectoderm, and embryonic lethality just prior to implantation (Gangloff et al., 2004; Murakami et al., 2004). By comparison, the effect of L-Gln does not depend on mTORC1 signaling (Figure 7) and results in translocation of p-Akt^S473^ but not p-ERK^T202/T204^ to the nucleus at the 2-cell stage (Figure 8). The effect of L-Gln on nuclear translocation at the 4- and 8-cell stages remains to be determined.

In addition, metabolic flux of L-Pro and L-Gln also play key roles in ESC fate including, in particular, through molecular mechanisms that alter the epigenetic landscape at both the histone and DNA levels (Comes et al., 2013; Carey et al., 2015; Ryu et al., 2015). To what extent these mechanisms play a role in improvements in pre-implantation development by L-Pro or L-Gln remains to be determined.

In LD culture under isosmotic conditions, development was significantly improved from the late 2-cell stage in the presence of either L-Pro or L-Gln (Figure 4). Furthermore, improved development was not observed if L-Pro was present only over the 1- to 2-cell stages (Figure 5). For L-Pro, the failure to improve development when it is only present at the 1- to 2-cell stages occurs despite the fact that it is taken up rapidly and to high (millimolar) concentration during this time via SIT1 (Anas et al., 2008). L-Pro may be rapidly effluxed once removed from the medium and therefore unable to mediate its effects at the post 2-cell stage. Consistent with this, the concentration of L-Gln in freshly isolated 2-cell mouse embryos rapidly becomes abnormally low when cultured in medium without L-Gln (Van Winkle and Dickinson, 1995). These results are consistent with those showing that, in general, non-essential amino acids (usually added in groups) improve *in vitro* development up to the ∼8-cell stage and reliance then “switches” to essential amino acids (Lane et al., 2001). After this time, developmental advantage in the presence of either L-Pro or L-Gln appear to be maintained rather than further improved (Figure 4). It is not known if this maintenance beyond the ∼8-cell stage in LD culture still requires the presence of these amino acids.

The competitive amino-acid inhibition profile observed here in terms of embryo development to the blastocyst stage (Figures 6A–C) showed that each of L-Leu, Gly and Betaine could inhibit the L-Pro-mediated effect. This inhibition profile does not fit that for the L-Pro transporter SIT1, which is not competitively inhibited by Gly (Anas et al., 2007), and which in any event is only expressed from 1- to 2-cell stage (Anas et al., 2008). Nor does the inhibition profile fit that for SNAT2, the transporter found in ESCs permitting L-Pro-mediated differentiation to primitive ectoderm (Lonic, 2007; Washington et al., 2010; Tan et al., 2011) as SNAT2 is not competitively inhibited by L-Leu (Tan et al., 2011). Instead, the profile fits that of a neutral amino-acid transporter; one option being B^0^AT1 (Slc6a19) (Böhmer et al., 2005; Bröer and Gether, 2012). Consistent with this, results from confocal microscopy using an anti-B^0^AT1 antibody show that this transporter is inserted into the plasma membrane at the 4-cell stage; i.e., during the late 2- to ∼8-cell stage window of development in which L-Pro improves development (Figures 4, 5; Day et al., 2013; Zada, Morris, Day unpublished).

The effect of amino acids in terms of hatching was different to that in terms of development to the blastocyst stage in two important respects: (i) L-Pro or L-Gln improved hatching not just in LD but also in HD culture (Figures 2Aii, 2Bii). (ii) L-Pro improvement in hatching was not inhibited by molar excess of Betaine. These results indicate that amino-acid-mediated improvement in hatching involves a different mechanism to that for improved development to the blastocyst stage. In particular, hatching does not appear to involve a mechanism involving autocrine/paracrine support or one mimicking autocrine/paracrine support. Furthermore, given the competitive inhibition profile is different, the transporter involved appears to be different. This is not surprising given that embryos dynamically express a range of amino-acid transporters in the pre-implantation stages (Van Winkle, 2001) suggesting they temporally exploit the use of different amino acids to promote key events in normal development (Shparberg et al., 2019b).

When groups of amino acids are added empirically to embryos cultured *in vitro* (Bavister and Arlotto, 1990; Gardner and Lane, 1993; Lane and Gardner, 1997; Lane et al., 2001; Summers and Biggers, 2003) competitive inhibition of uptake may mean that the presence of one type of amino acid mitigates the positive effects on development of one or more others. Our data reinforce the idea that rational design of improved culture media for embryos depends on quantitative information involving a number of factors, including: Identification of amino acids which have a positive effect and the optimal concentrations to use, the time in development to add and withdraw them, and elimination of amino acids which act as competitors (or at least consideration of the molar ratios to use). The last of these can be refined by understanding the temporal expression of specific amino-acid transporters (Van Winkle, 2001; Pelland et al., 2009), together with their relative rates of transport and inhibitory constants. *In vivo*, this optimization is, presumably, evolutionarily well established and thus information on the composition of reproductive-tract fluid and its changing composition both over time and along the tract itself would also be instructive (Miller and Schultz, 1987; Van Winkle and Dickinson, 1995; Elhassan et al., 2001; Aguilar and Reyley, 2005; Li et al., 2006).

## Data Availability Statement

All datasets generated for this study are included in the article/supplementary material.

## Ethics Statement

The animal study was reviewed and approved by University of Sydney Animal Care and Ethics Committee (approval numbers 4838, 5583, and 824).

## Author Contributions

MM and MD were responsible for writing and preparing the drafts and final manuscript. MD designed and prepared the figures, with legends, based on the data generated by SO, MatZ, MarZ, and RZ. MM, MD, and MT conceived the project and designed the original experiments. SO was the principal contributor of data for this research. All authors read, edited and approved the final manuscript.

## Conflict of Interest

The authors declare that the research was conducted in the absence of any commercial or financial relationships that could be construed as a potential conflict of interest.
